# Contributions of expected sensory and affective action effects to action selection and performance: Evidence from forced- and free-choice tasks

**DOI:** 10.3758/s13423-016-1139-x

**Published:** 2016-08-12

**Authors:** Bernhard Hommel, Dominique P. Lippelt, Ermine Gurbuz, Roland Pfister

**Affiliations:** 1 Cognitive Psychology Unit & Leiden Institute for Brain and Cognition, Leiden University, Leiden, The Netherlands; 2 Institute for Psychological Research, Cognitive Psychology Unit, Leiden University, Wassenaarseweg 52, 2333 AK Leiden, The Netherlands; 3 Department of Psychology III, University of Würzburg, Würzburg, Germany

**Keywords:** Cognitive control, Automaticity, Motor planning/programming, Stimulus–response compatibility, Emotion

## Abstract

Whereas ideomotor approaches to action control emphasize the importance of sensory action effects for action selection, motivational approaches emphasize the role of affective action effects. We used a game-like experimental setup to directly compare the roles of sensory and affective action effects in selecting and performing reaching actions in forced- and free-choice tasks. The two kinds of action effects did not interact. Action selection and execution in the forced-choice task were strongly impacted by the spatial compatibility between actions and the expected sensory action effects, whereas the free-choice task was hardly affected. In contrast, action execution, but not selection, in both tasks was strongly impacted by the spatial compatibility between actions and highly valued action effects. This pattern suggests that sensory and affective action effects serve different purposes: The former seem to dominate rule-based action selection, whereas the latter might serve to reduce any remaining action uncertainty.

**Electronic supplementary material:**

The online version of this article (doi:10.3758/s13423-016-1139-x) contains supplementary material, which is available to authorized users.

Human actions are taken to reflect the goals that the actor intends to achieve. How such goals impact action control remains an open issue, especially because the dominant theoretical approaches currently emphasize different aspects of goal achievement. The *ideomotor approach* to intentional action holds that agents register the sensory consequences of their actions and automatically integrate the representations of these consequences with the motor patterns producing them (Shin, Proctor, & Capaldi, 2010). The resulting associations are assumed to be bidirectional, so that later action selection can reactivate the required motor patterns by reactivating representations of the to-be-expected sensory action effects (e.g., through imagining). In contrast, the *motivational approach* focuses not on the sensory effects of actions, but rather on the affective states they create. Actions resulting in positive affective states are assumed more likely to be selected than those resulting in negative states (de Wit & Dickinson, 2009).

The two approaches agree in emphasizing the perceptual (i.e., sensory or affective) outcomes of actions, and they both have received ample empirical support over the last decades, but very little is known about how representations of the expected sensory and affective outcomes of actions contribute or interact to support action control. In a recent study, Eder, Rothermund, de Houwer, and Hommel (2015) investigated what they called the “directive” and “incentive” functions of affective action effects, by means of an action–effect learning paradigm (Elsner & Hommel, 2001). Their participants first learned to produce a pleasant action effect with one action and an unpleasant effect with another. In a subsequent test phase, the same actions were carried out in response to a neutral feature of affective stimuli. The findings revealed that responses were faster when the irrelevant valence of the stimulus matched the valence of the response outcome. This suggests that representations of the affective action effects were involved in action control by promoting selection of the actions that were expected to produce the same effect. Interestingly, this effect was the same for both pleasant and unpleasant responses, which indicates that the effect was directive and not motivational in nature. However, when the test was carried out with a free-choice task, a clear motivational effect was obtained: Actions with pleasant effects were more often selected than actions with unpleasant effects. Along the same lines, Watson, Wiers, Hommel, and de Wit (2014) found that satiation on food reward (either Smarties or popcorn) reduced responding on the key associated with this food in a free-choice task that included no food-associated cues. Interestingly, however, satiation failed to reduce cue-elicited food-seeking when food-associated stimuli were presented in a transfer test. Taken together, these two studies converge in suggesting that, if both sensory and affective action consequences are varied, the expected sensory consequences drive action selection in forced-choice tasks, whereas the expected affective consequences drive action selection in free-choice tasks.

The aim of the present study was to extend the analysis of the roles of sensory and affective action effects to currently unexplored parameters relating to the movement trajectories of reaching actions. Importantly for our purposes, Pfister, Janczyk, Wirth, Dignath, and Kunde (2014) proposed a game-like setup to investigate the impact of sensory action effects on the kinematics of such reaching actions. Participants moved an avatar by means of a computer mouse from a neutral position toward either the left or the right side of a screen. The avatar would enter a portal that would displace it to the final location, on the same or the opposite side of the screen. The results showed that the moving hand was systematically attracted toward the eventual final location, which demonstrates that this location was actively represented during the action. Furthermore, Dignath, Pfister, Eder, Kiesel, and Kunde (2014) reported a similar observation for affective action effects: Movements toward negatively rewarded objects showed systematic deviations toward positively rewarded objects, even if none of the rewards was visible during the reaching action. Hence, both anticipated sensory and anticipated affective action effects have been demonstrated to leave their fingerprints on the trajectories of reaching responses, even though the action effects were not present during action planning and execution.

In the present study, we used the same experimental design, but we combined sensory and affective action effects to study their interplay during action selection and the following movement trajectories. Note that this distinction is not meant to imply discrete processes in terms of planning versus online control; rather, we intended to study the impact of anticipated action effects on movement trajectories, regardless of whether the impact originated early during planning or later during online control. We further contrasted a forced-choice task, in which participants were told which of two actions to execute, and a free-choice task in which they could decide between the two alternatives. In particular, we had participants move an avatar to a portal on the left or the right side of a screen in order to collect a cake of high or low value (negative values were not used, to prevent avoidance-related processes; Eder & Hommel, 2013). The cakes were invisible until the end of the action but always stayed in the same location on the left or the right of the screen. Moreover, the cakes could not be accessed directly, but rather through a portal on the same or the opposite side, as indicated by the status of the portal.

This setup allowed for orthogonally manipulating the two action–effect relationships. For one thing, the location of the cake (the actual action goal) was either spatially compatible or incompatible with the action (i.e., the location to which the avatar was to be moved), depending on the status of the portal. This sort of action–effect compatibility (AEC) was considered to tap into the impact of sensory action effects, and a compatible relation was expected to yield better performance (Pfister et al., 2014; Wirth, Pfister, Janczyk, & Kunde, 2015). For another, the targeted cake was either low or high in value. This allowed for the manipulation of affective AEC, since the higher-valued cake (which should induce more approach motivation) was either spatially compatible or spatially incompatible with the action. Again, the compatible condition was expected to yield better performance (Dignath et al., 2014).

In this setting, both kinds of AEC referred to events that were not present during action execution. Thus, if AEC were to have an effect on any of the measures (reaction times [RTs], movement times [MTs], or trajectory deviations), this would imply that action control processes are affected by and/or operate on representations of action effects (i.e., possible sensory or affective action goals). In most trials (67 %), a stimulus would indicate the cake to be collected, which rendered the task a forced-choice task. In the remaining trials (33 %), the stimulus would leave the choice to the participant, which rendered the task a free-choice task. Given the findings of Watson et al. (2014) and Eder et al. (2015), one would expect that sensory and affective forms of AEC would impact action control in an additive fashion, but that sensory effects might dominate in the forced-choice task and affective effects dominate in the free-choice task. We were particularly interested to see whether this pattern of results would emerge for MTs and trajectory deviations, which would indicate an impact on action execution.

## Method

### Participants

We recruited 45 participants, of whom four were excluded from further analysis because of high error rates (>20 %) on forced-choice trials, and a further six were excluded because they did not go for the low reward on any of the free-choice trials (i.e., they chose the high reward in all free-choice trials, leading to empty design cells). The mean age for the remaining 35 participants (24 females, 11 males) was 21.7 years (*SD* = 2.78). Participants received a combination of course credit and a small amount of money that depended on their performance (€3.50–€5.50, depending on performance). All participants were naive regarding the hypotheses of the experiment.

## Materials

**Fig. 1:**
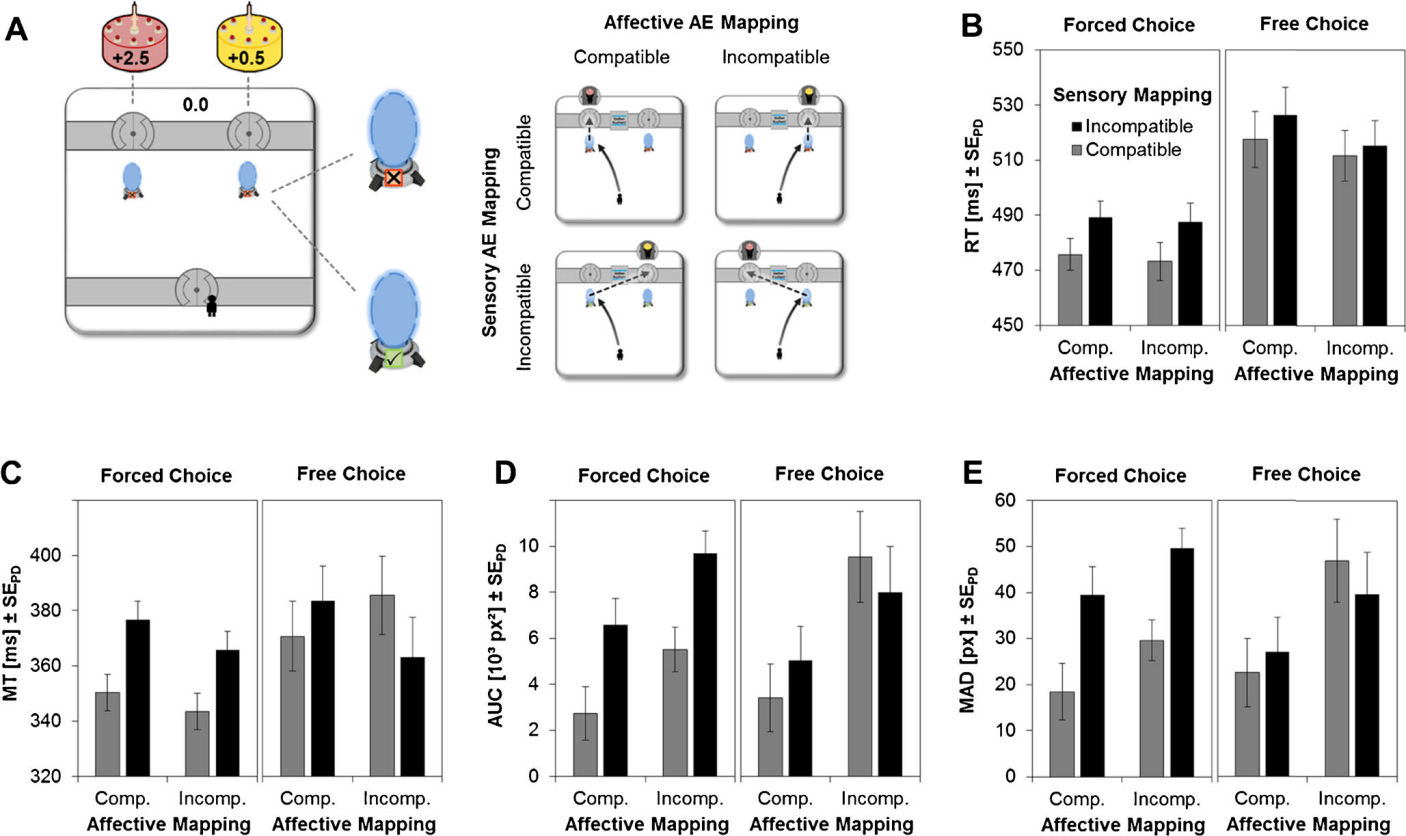
Experimental design and results. (**a**) Participants controlled a virtual avatar by moving the computer mouse. In each trial, they collected a cake that yielded either high or low reward (2.5 vs. 0.5 eurocents). The cakes were located behind a left or a right door, and the doors were accessed via portals that teleported the avatar either to the adjacent door (portals off) or to the door on the opposite side of the display (portals on). This allowed us to manipulate affective action–effect compatibility (AEC) and sensory AEC independently from one another: Movements toward the high- versus the low-reward cake implied compatible versus incompatible affective AEC relations, whereas portals that were switched off versus on implied compatible versus incompatible sensory AEC relations. (**b**–**e**) The remaining panels show reaction times (RT; **b**), movement times (MT; **c**), areas under the curve (AUC; **d**), and maximum absolute distances (MAD; **e**) as a function of affective and sensory forms of AEC. Results are shown separately for forced-choice and free-choice actions. *Error bars* indicate standard errors of the paired differences (*SE*_PD_; Pfister & Janczyk, 2013), computed separately for each sensory AEC effect

The stimuli used in the experiment were adapted from the computer game *Portal* (www.thinkingwithportals.com; see Fig. 1a). All stimuli were presented on a 16-in. screen with a resolution of 1,024 × 768, running at 60 Hz. The stimulus screen consisted of two walls (height = 1.8 cm), each distanced about 4.2 cm from the wall midline to the upper and lower borders of the screen, respectively, resulting in a distance of 14 cm between the walls. The lower wall contained one door in the middle (2.5 cm × 2.2 cm), whereas the upper wall had two doors located 8.3 cm from the left and right screen borders. In front of the two upper doors were two portals (1.3 cm × 2.4 cm), appearing 2.1 cm below each door. These portals could be marked with either a cross on a red background or a check mark on a green background, indicating the status of the portal. The distance between the start position and each of the portals was approximately 13.7 cm. Participants operated a standard computer mouse, and the mouse cursor was replaced by a schematic avatar (0.7 cm × 1.5 cm).

Imperative stimuli appeared in the center of the upper wall (2.5 cm × 2.8 cm) as soon as the lower door was opened by the participant. Either an imperative stimulus required the participants to collect one or the other virtual cake (“Red Cake!” or “Yellow Cake!”), or the choice was left up to the participant (“Choose a cake!”). At the top center of the screen a small counter was presented, displaying how much money the participant had won so far.

Before the experimental task, participants further completed the English version of the Behavioral Inhibition/Approach System (BIS/BAS) to assess their dispositional BIS and BAS sensitivities (Carver & White, 1994). For the present study, we were interested only in the Responsiveness to Reward subscale of this questionnaire (see Muhle-Karbe & Krebs, 2012, for a similar approach).

### Procedure

Participants went through a short tutorial that familiarized them with the setup and explained the meanings of the different elements. The participants were instructed that each door would reveal either a red or a yellow cake (counterbalanced across participants), which they were to collect depending on the stimulus. They were also informed that the locations of the cakes would not change throughout the experiment. They learned that they would have to use the portals to teleport an avatar to the cake locations, but that the portals could be turned on (signaling teleporting to the opposite side), indicated by a checkmark on a green background, or off (signaling teleporting to the same side), indicated by a cross on a red background. Participants were told that they could earn a small amount of money for each cake they collected, but that one color (counterbalanced) was worth 0.5 eurocents, and the other 2.5 eurocents, per cake. They were encouraged to collect the low- and high-reward cakes with about equal frequencies on free-choice trials, but it was explained that they were not obliged to do so.

Before each trial the avatar appeared below the lower wall, and participants were given time to check the portal status. To start the actual trial, they moved the avatar to the front door and waited for 500 ms. Then the door opened, and an imperative stimulus appeared in the center of the upper wall instructed the participant which cake to collect. Participants had to collect the cake within 2,000 ms or they were presented with a feedback message that they were too slow. From the moment that the imperative stimulus appeared and onward, the cursor position was recorded until the end of the trial, with a sampling rate of at least 250 Hz (depending on current CPU load).

Upon reaching a portal, the avatar was teleported to the corresponding door. If participants collected the correct cake, they saw their avatar with a happy face and the cake in its hands, but collecting the incorrect cake resulted in a sad-looking avatar. Additionally, collecting the correct cake added the amount of earned money to a counter presented in the top center of the screen. This feedback stayed on screen for 2,000 ms, and mouse movements no longer affected the display. Finally the display was cleared, and the next trial started after 1,000 ms.

Participants went through one practice block and four experimental blocks of 60 trials each. Each block was followed by a short break that provided participants with feedback regarding their average RT, number of errors, and how much money they had won up to that point.

### Data treatment

RTs were measured from the presentation of the imperative stimulus until the avatar had left its starting position, whereas MTs were measured from that point until the cursor hit the border of a portal. Trajectory data were analyzed offline after time normalization to 101 steps, with movements to the left being mirrored on the vertical axis to allow for aggregation across both movement directions (for details, see Pfister et al., 2014). From the interpolated data, we computed the (maximum) absolute distances (MADs) and areas under the curve (AUCs) relative to a straight line from start to endpoint of the movement. Deviations away from the targeted portal were counted as positive values, and we expected the MADs and AUCs to be affected in similar ways. We chose to analyze both (ideally converging) measures in order to overcome possible pitfalls of the individual measures, with MAD being influenced more heavily by outliers within a trajectory, and AUC being influenced by possible compensating movements after a trajectory deviation (see Pfister, Wirth, Schwarz, Steinhauser, & Kunde, 2016).

For all analyses, we removed trials with errors (3.7 %) and trials following errors. We further discarded all trials as outliers if any measure deviated from the respective cell mean by more than 2.5 standard deviations (4.8 %). All four variables were analyzed by means of 2 (Task: free vs. forced choice) × 2 (Sensory AEC) × 2 (Affective AEC) analyses of variance (ANOVAs) for repeated measures (see the Supplementary Material, Tables S1 and S2, for descriptive statistics and a detailed list of all ANOVA tables).

### Hypotheses

We tested three key hypotheses. First, we expected sensory AEC to have a stronger effect in the forced-choice than in the free-choice task, which would be reflected in significant interactions of sensory AEC and task. Second, we expected affective AEC to have a strong impact on the free-choice task (though not necessarily more pronounced than in the forced-choice task), which would be reflected in either a significant main effect of affective AEC or an interaction of affective AEC and task. Finally, we predicted that sensory AEC and affective AEC would combine in an additive fashion; that is, we predicted that the corresponding interaction would return nonsignificant results. As was outlined in the introduction, we were mainly interested in the three postinitiation measures (MT, MAD, and AUC) for all three hypotheses.

## Results

The mean RTs and MTs for the eight conditions are shown in Fig. 1b and c, and d and e show the corresponding AUC and MAD results. In the free-choice task, the higher-valued cake was chosen more often than the lower-valued cake (64 vs. 36 %; cf. Watson et al., 2014), indicating that the different rewards did induce corresponding motivational tendencies (note that these numbers do not include participants who went exclusively for the higher-valued cake).

Sensory AEC affected all four measures, indicating longer RTs, *F*(1, 34) = 6.51, *p* = .015, *η*_p_^2^ = .161, and MTs, *F*(1, 34) = 5.91, *p* = .020, *η*_p_^2^ = .148, and more pronounced AUC, *F*(1, 34) = 5.56, *p* = .024, *η*_p_^2^ = .140, and MAD, *F*(1, 34) = 5.89, *p* = .021, *η*_p_^2^ = .148, for incompatible than for compatible trials. For the three postinitiation measures (MT, AUC, and MAD), this effect was modified by task, indicating that (as separate ANOVAs confirmed), the sensory AEC effect was restricted to the forced-choice task, and not significant in the free-choice task: MT, *F*(1, 34) = 10.16, *p* = .003, *η*_p_^2^ = .230; AUC, *F*(1, 34) = 8.66, *p* = .006, *η*_p_^2^ = .203; MAD, *F*(1, 34) = 11.34, *p* = .002, *η*_p_^2^ = .250. Descriptively, a similar pattern emerged for RTs, though the interaction of AEC and task failed to reach significance for this measure, *F*(1, 34) = 1.08, *p* = .306, *η*_p_^2^ = .031.

Affective AEC produced main effects on AUC, *F*(1, 34) = 8.83, *p* = .005, *η*_p_^2^ = .206, and MAD, *F*(1, 34) = 7.18, *p* = .011, *η*_p_^2^ = .174, indicating more deviations toward the higher-valued cake. This effect was not modified by task, *p*s > .25 (note that in the free-choice task, high-reward trials were more frequent than low-reward trials). We observed no interaction of sensory and affective AEC for either measure, *p*s > .126 (see Table S2 in the Supplementary Material for all four relevant ANOVA tables).

Finally, main effects of task indicated that both RTs, *F*(1, 34) = 40.00, *p* < .001, *η*_p_^2^ = .540, and MTs, *F*(1, 34) = 5.41, *p* = .026, *η*_p_^2^ = .137, were longer for free- than for forced-choice trials—a common finding (Berlyne, 1960). We did not find any significant correlations between reward responsiveness and any of the effects described above.

## Discussion

Three findings are of particular relevance. First, we found no indication of any direct interaction between sensory and affective AEC, which supports the assumption of Eder et al. (2015) that sensory and affective action effects impact action control in an additive fashion.

Second, the impact of sensory AEC was mainly (or, in the postselection measures, exclusively) present in the forced-choice task. This is consistent with the observation of Watson et al. (2014) that cues related to the sensory representations of action goals have a stronger impact on stimulus-driven decision-making, where they can fully compensate for the loss of any motivational support for an action outcome (as through satiation). It is interesting to see that this impact goes beyond action selection, but also keeps pulling the action toward the location of the intended outcome (Pfister et al., 2014). This observation is in line with previous observations on the influence of anticipated auditory action effects on the accuracy and trajectory of sequential movements in timing tasks (Keller & Koch, 2006; Keller, Dalla Bella, & Koch, 2010). Both findings suggest that action selection is not a discrete process with a defined ending, which fits with ideomotor ideas that selecting an action implies a bias toward its execution, without necessarily stopping competition from alternative actions (Hommel, 2009). Rather, the conflict between alternative goals seems to stay active until the action is completed, which is consistent with the observation that stimulus–response compatibility effects can be obtained even in the absence of any response uncertainty (Hommel, 1996). It is also worth noting that sensory AEC was manipulated in a trial-to-trial fashion in the present design, adding to the growing body of evidence suggesting that corresponding effect anticipations can be controlled flexibly to account for the current context (Ansorge, 2002; Gaschler & Nattkemper, 2012; Pfister, Kiesel, & Melcher, 2010; Zwosta, Ruge, & Wolfensteller, 2013).

Third, affective AEC had no impact on the temporal variables (RT, MT), but only on the spatial attraction of the corresponding trajectories. We consider the first part of this observation less interesting than the second part, because RT effects may well be obtained under conditions that (a) optimize the experimental design toward this measure and (b) render affective outcomes more salient—such as by using negative affective effects (Beckers, De Houwer, & Eelen, 2002; Eder et al., 2015). Importantly, however, the second part indicates that both tasks were affected alike, suggesting that affective action effects exerted a robust attraction effect on behavior—much as they did in the study by Dignath et al. (2014). The independence of sensory and affective effects further suggests that these effects play different roles in action control. If we assume that representations of sensory action effects serve to select and maintain the actions that realize intended outcomes, the role of affective action effects may consist in (a) reducing uncertainty that sensory effects cannot eliminate, and/or (b) choosing the most “attractive” (Damasio, Tranel, & Damasio, 1991) or energy-saving (Rosenbaum, Meulenbroek, Vaughan, & Jansen, 2001) among multiple equally suited action alternatives. Considering that sensory action effects commonly underspecify the precise kinematics of actions and that free-choice tasks include more uncertainty than forced-choice tasks, this division of labor would account for our present findings.

The assumption of independent mechanisms for sensory and affective action effects also seems to fit well with recent findings on adaptation to cognitive and affective conflict, respectively. More often than not, such studies have reported absent across-task adaptation effects between cognitive conflict (as evoked in the Simon task) and affective conflict (as evoked by valence-based interruption; Kunde, Augst, & Kleinsorge, 2012; Wirth, Pfister, & Kunde, 2016), even in settings that yielded reliable across-task adaptation effects for two different cognitive conflict tasks (Funes, Lupiáñez, & Humphreys, 2010; Torres-Quesada, Funes, & Lupiáñez, 2013). At the same time, however, adaptation to cognitive conflict has been shown to be susceptible to general emotional states (van Steenbergen, Band, & Hommel 2009, 2010). This might be taken to suggest that more sustained emotional states, such as one’s current mood or reward context, might yield a corresponding pattern of results regarding the processing of sensory action effects. Results in accordance with this speculation can indeed be found in the literature (Muhle-Karbe & Krebs, 2012), though this issue certainly awaits further investigation.

## Electronic supplementary material

(DOCX 23.4 kb)
