# Supplementary material for: Contributions of expected sensory and affective action effects to action selection and performance: Evidence from forced- and free-choice tasks
Source: Psychon Bull Rev. 2016 Aug 12;24(3):821–7. doi: 10.3758/s13423-016-1139-x (PMC5486880; doi:10.3758/s13423-016-1139-x)
Supplement: Supplementary file 1 — (DOCX 23.4 kb) [file 13423_2016_1139_MOESM1_ESM.docx]

**Supplementary Material for**

**Contributions of expected sensory and affective action effects to action selection and performance: evidence from forced- and free-choice tasks**

Descriptive statistics for all dependent variables can be found in Table S1. These statistics are accompanied by a full report of the corresponding analyses of variance (ANOVAs), as displayed in Table S2, ae we only referred to theoretically informative effects in the main text for reasons of brevity. Finally, Table S3 lists descriptive statistics for percentages of errors (PEs) in forced-choice trials. Please note that these results are based on commission errors only, whereas other errors – such response anticipations – were removed prior to calculating PEs. No effect was significant when analyzing PEs by means of a 2 (sensory action-effect compatibility, AEC) x 2 (affective AEC) ANOVA, *F*s < 1.21, *p*s > .279, indicating that the main results of the experiment were not due to speed-accuracy trade-offs.

**Tab. S1.** Descriptive statistics for all four measures, i.e., reaction times (RTs, in ms), movement times (MT, in ms), areas under the curve (AUCs, in px²), and maximum absolute distances (MADs, in px).

| **Measure** | **Sensory Mapping** | **Task** | | | |
| --- | --- | --- | --- | --- | --- |
|  |  | Forced Choice | | Free Choice | |
|  |  | **Affective Mapping** | | **Affective Mapping** | |
|  |  | Compatible | Incompatible | Compatible | Incompatible |
| RT | Incompatible | 489 | 488 | 526 | 515 |
|  | Compatible | 476 | 473 | 518 | 512 |
| MT | Incompatible | 377 | 366 | 383 | 363 |
|  | Compatible | 350 | 343 | 371 | 385 |
| AUC | Incompatible | 6560 | 9695 | 5024 | 7983 |
|  | Compatible | 2722 | 5506 | 3400 | 9537 |
| MAD | Incompatible | 39 | 50 | 27 | 40 |
|  | Compatible | 18 | 30 | 23 | 47 |

**Tab. S2.** Results tables of each analysis of variance (ANOVA) with the factors sensory action-effect compatibility (AEC; compatible vs. incompatible), affective AEC, and task (forced-choice vs. free-choice). Measures are labelled as in Table S1.

| **Measure** | **Source of variance** | **F** | **p** | **η_p_^2^** |
| --- | --- | --- | --- | --- |
| RT | Sensory AEC | 6.51 | .015 | .161 |
|  | Affective AEC | 1.83 | .185 | .051 |
|  | Task | 39.99 | <.001 | .540 |
|  | Sensory AEC * Affective AEC | 0.07 | .793 | .002 |
|  | Sensory AEC * Task | 1.08 | .306 | .031 |
|  | Affective AEC * Task | 1.15 | .290 | .033 |
|  | Three-way interaction | 0.12 | .735 | .003 |
| MT | Sensory AEC | 5.91 | .020 | .148 |
|  | Affective AEC | 1.08 | .307 | .031 |
|  | Task | 5.41 | .026 | .137 |
|  | Sensory AEC * Affective AEC | 2.46 | .126 | .067 |
|  | Sensory AEC * Task | 10.16 | .003 | .230 |
|  | Affective AEC * Task | 0.52 | .475 | .015 |
|  | Three-way interaction | 1.63 | .210 | .046 |
| AUC | Sensory AEC | 5.56 | .024 | .140 |
|  | Affective AEC | 8.83 | .005 | .206 |
|  | Task | 0.19 | .667 | .005 |
|  | Sensory AEC * Affective AEC | 1.00 | .324 | .029 |
|  | Sensory AEC * Task | 8.66 | .006 | .203 |
|  | Affective AEC * Task | 1.35 | .254 | .038 |
|  | Three-way interaction | 1.91 | .176 | .053 |
| MAD | Sensory AEC | 5.89 | .021 | .148 |
|  | Affective AEC | 7.18 | .011 | .174 |
|  | Task | 0.00 | .962 | .000 |
|  | Sensory AEC * Affective AEC | 0.90 | .350 | .026 |
|  | Sensory AEC * Task | 11.34 | .002 | .250 |
|  | Affective AEC * Task | 1.26 | .270 | .036 |
|  | Three-way interaction | 0.64 | .428 | .019 |

**Tab. S3.** Percentage errors (PEs) for each condition of the forced-choice task.

| **Sensory Mapping** | **Affective Mapping** | |
| --- | --- | --- |
|  | Compatible | Incompatible |
| Incompatible | 3.02 | 3.09 |
| Compatible | 2.75 | 2.34 |
